# The Interaction among Microbiota, Immunity, and Genetic and Dietary Factors Is the *Condicio Sine Qua Non* Celiac Disease Can Develop

**DOI:** 10.1155/2015/123653

**Published:** 2015-05-18

**Authors:** D. Pagliari, R. Urgesi, S. Frosali, M. E. Riccioni, E. E. Newton, R. Landolfi, F. Pandolfi, R. Cianci

**Affiliations:** ^1^Institute of Internal Medicine, Catholic University, 00168 Rome, Italy; ^2^Gastroenterology and Digestive Endoscopy Unit, Belcolle Hospital, 01100 Viterbo, Italy; ^3^Digestive Endoscopy Unit, Catholic University, 00168 Rome, Italy; ^4^CytoCure LLC, Beverly, MA 01915, USA

## Abstract

Celiac disease (CD) is an immune-mediated enteropathy, triggered by dietary wheat gluten and similar proteins of barley and rye in genetically susceptible individuals. This is a complex disorder involving both environmental and immune-genetic factors. The major genetic risk factor for CD is determined by HLA-DQ genes. Dysfunction of the innate and adaptive immune systems can conceivably cause impairment of mucosal barrier function and development of localized or systemic inflammatory and autoimmune processes. Exposure to gluten is the main environmental trigger responsible for the signs and symptoms of the disease, but exposure to gluten does not fully explain the manifestation of CD. Thus, both genetic determination and environmental exposure to gluten are necessary for the full manifestation of CD; neither of them is sufficient alone. Epidemiological and clinical data suggest that other environmental factors, including infections, alterations in the intestinal microbiota composition, and early feeding practices, might also play a role in disease development. Thus, this interaction is the *condicio sine qua non* celiac disease can develop. The breakdown of the interaction among microbiota, innate immunity, and genetic and dietary factors leads to disruption of homeostasis and inflammation; and tissue damage occurs. Focusing attention on this interaction and its breakdown may allow a better understanding of the CD pathogenesis and lead to novel translational avenues for preventing and treating this widespread disease.

## 1. Introduction

Celiac disease (CD) is an immune-based enteropathy triggered by dietary wheat gluten and similar proteins in barley and rye in genetically susceptible individuals. Recently, the ESPGHN (European Society of Paediatric Gastroenterology, Hepatology and Nutrition) proposed that CD may be defined as an immune-mediated systemic disorder elicited by gluten and related prolamins in genetically susceptible individuals and characterized by a variable combination of gluten-dependent manifestations, CD-specific antibodies, and HLA-DQ2 or HLA-DQ8 haplotypes, causing duodenal chronic inflammation [1]. CD is a T-cell mediated disease, in which gliadin-derived peptides activate lamina propria infiltrating T lymphocytes [2]. These latter represent the pivotal cells that orchestrate tissue damage. This leads to the release of proinflammatory cytokines, such as IFN-*γ* and IL-15 [3] which are responsible for the activation of the cytotoxic activity of intraepithelial lymphocytes (IELs) that leads to a profound tissue remodeling [4]. This is a complex disorder, with environmental and immune-genetic factors contributing to its etiology. The main genetic influence on CD is the HLA locus [5], specifically MHC class II genes that encode HLA-DQ2 (HLA-DQ2.5 and HLA-DQ2.2) and HLA-DQ8 heterodimers. The prevalence of disease is usually reported to be about 1% in the general US population [6] but there are emerging data suggesting an increase in some countries. Although there is a strong genetic predisposition, resulting from the presence of the HLA gene DQ2/DQ8 in the development of CD, and gluten is the main environmental factor responsible for the signs and symptoms of this disorder, neither genetic nor environmental factors show 100% correlation. Thus other immune, genetic, and environmental factors must be involved in CD onset [7].

## 2. Pathogenesis and Pathogenic Model of Celiac Disease

CD is considered an autoimmune disorder with both genetic and environmental components. Evidence for a genetic component is best exemplified by the strong dependence on the presence of the HLA-DQ2 (encoded by the alleles DQA1^∗^05 and DQB1^∗^02) and HLA-DQ8 (DQA1^∗^03 and DQB1-^∗^0302) haplotypes. More than 95% of those with CD express HLADQ2 while the rest express HLA-DQ8. However, about 30–40% of the general population expresses HLA-DQ2, so while these HLA genes are necessary, they are not sufficient for developing disease and clearly non-HLA genes are also involved. At least 39 non-HLA genes have been identified through genome-wide association studies as strongly associated with CD [8].

Most of these genes are involved in control of the innate and adaptive immune response.

However, the role of these genes in the development of CD is not completely clear. The primary environmental trigger is gluten, the protein fractions of wheat, barley, and rye. The incomplete digestion of this protein by humans, due to high concentrations of glutamine and proline, results in the formation of residual partially digested peptides. These peptides are responsible for innate and adaptive immune responses underlying the disease in genetically predisposed subjects. Other trigger factors may play a role in precipitating disease. Currently, some studies are evaluating the role of the modifications of gut microbiota as a factor contributing to the onset of disease [9].

## 3. Immunopathogenesis

Although the precise immune mechanisms that are involved in the progressive destruction of the small intestinal mucosa remain to be elucidated, the hallmark of CD is an immune-mediated enteropathy that involves both of the innate and adaptive immune system. In relation to innate immune system response, we should consider that some gluten peptides can induce tissue damage by directly activating components of innate immunity [10]. The peptide (*α*-gliadin 31-43) p31-43/49 has been shown to activate the production of IL-15 and the NK receptor-mediated cytotoxicity by IELs [11, 12], independent of TCR specificity, and to induce apoptosis of enterocytes, upregulate MHC class I molecules, activate MAP kinase pathway, and upregulate the expression of CD83, a maturation marker of dendritic cells [13]. The presence of a receptor for p31-43/49 in intestinal epithelial cells has not been found yet and, thus, the molecular mechanism underlying the biological effects observed as a consequence of the interaction of this peptide with the gut mucosa remains still unclear [14]. The DQ2 and DQ8 molecules confer susceptibility to CD by presenting disease-related peptides to T-cells in the small intestine or by shaping the T-cell repertoire during T-cell development in the thymus. Initially, paracellular passage of gliadin peptides is due to an increase of gut permeability which, in turn, is due to an upregulation of zonulin, an intestinal peptide involved in epithelial tight junction control [15]. Also, IL-15 contributes to promoting the CD4+ T-cell adaptive immune response [3, 16]. IL-15 up-regulates both CD94/NKG2C and NKG2D NK receptors boosting their ability to lyse enterocytes [14]. The adaptive immune response in CD is characterized predominantly by production of the proinflammatory cytokine interferon-*γ* (IFN-*γ*) from gluten-specific CD4+ T-helper cells triggered by gluten-derived peptides recognized by HLA-DQ2 or HLA-DQ8 heterodimers of antigen-presenting cells (APCs) [17]. Tissue transglutaminase-2 (TTG2) converts noncharged glutamine into negatively charged glutamic acid through a process called deamidation. In fact, the peptide-binding motifs of DQ2 and DQ8 predict a preference for negative charges at anchor positions of the bound peptides. DQ2 has a preference for negatively charged residues at the P4, P6, and P7 anchor positions [18], whereas DQ8 has a preference for negatively charged residues at anchor positions P1, P4, and P9 [19]. Generally, gluten proteins contain few negatively charged residues but in active CD the level of expression of enzyme TG2 is increased and the ratio of deamidation to transamidation is markedly increased. In active CD TG2 is expressed at the epithelial brush border, as well as being expressed extracellularly in the subepithelial region. The pH in the proximal small intestine is ~6.6 which should allow marked TG2-mediated deamidation of peptides in the brush border [20]. This process increases the negative charge on the peptide molecule and enhances binding of the peptide within the peptide binding groove of the HLA-DQ2 molecule on the surface of the APCs. This is a prerequisite for a gluten-specific T-cell response as well as a B-cell response that results in production of the anti-TTG antibodies that represents an epiphenomenon of the CD pathogenesis and may be used in the diagnostic pathway [15, 21]. Furthermore, deamidated gluten peptides are presented to CD4+ T-cells that release proinflammatory cytokines activating cytotoxic CD8 IELs (CD8+ TCR*αβ*+ and TCR*γδ*+ T-cells) contributing to a profound tissue remodeling and damage [13, 22]. While IgA antibodies against either gluten or the autoantigen TG2 serve as a highly useful means of testing for CD, their precise role in the immunopathogenesis of the condition remains yet unknown. Whether they are byproducts of the intestinal adaptive immune response or they play a direct role in CD pathogenesis remains unclear. There is evidence that IL-21 plays a role in the pathogenesis of CD as high levels of this cytokine can be demonstrated in biopsies from those with active disease that are not on treatment [23]. However, the mechanism whereby IL-21 is produced and the precise role it plays in the disease process remains unexplained. More recently, it has been proposed that, in the intestine of CD subjects, normal gluten peptides may be complexed to intraluminal secretory IgA, bound to an IgA receptor and transported, and protected from lysosomal degradation by a specific transcytosis pathway involving the transferrin receptor CD71. Compared to nonceliac subjects or CD subjects on a gluten-free diet, CD71 expression is increased in CD sufferers and colocalizes with IgA at the apical enterocyte membrane [24].

**Figure 1:**
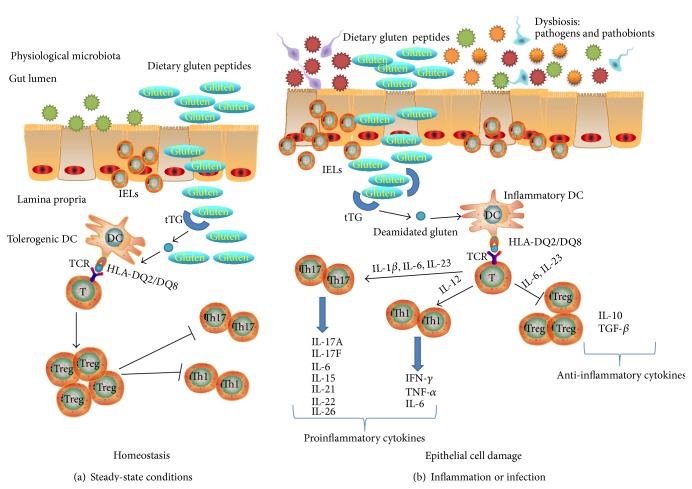
The complex interconnection among immune system, microbiota, and environmental factors (including dietary food antigens and/or infection) in the pathogenesis of celiac disease. (a) Steady-state condition. Dietary antigens and physiologic microbiota are in symbiotic relationship with host mucosal cells; thereby a harmonized balance between pro- and anti-inflammatory factors is achieved (homeostasis). (b) Inflammation or infection. The breakdown of the normal microbial community contributes to dysbiosis. In CD patients, gluten derived peptides are recognized by antigen presenting cells, with T-cells response. Deaminated gluten peptides are presented to T-cells with subsequent release of proinflammatory cytokines. In this way, Tregs are suppressed. This fact leads to a break of balance with consequent epithelial cell damage.

In summary, the disease develops as a result of an abnormal CD4+ T-cell-initiated immune response to gluten. Generally, gluten gains access to Peyer's patches physiologically via M cells or supraphysiologically during periods of increased epithelial permeability [25]; thus, it is processed by Peyer's patches dendritic cells [26] and presented to CD4+ T-cell [27]. In nonceliac subjects the presentation of gluten peptides on HLA-DQ2/DQ8 induces a Th2 response [28]; in celiac subjects in the presence of TTG2 activity and deamidation of gluten peptides in the APCs the response is biased towards Th1 response [29]. Presentation of gluten to T-cells could be carried out not only by dendritic cells but also by macrophages, B-cells, and even enterocytes that express HLA class II [30]. Enterocytes can present antigens to lipoprotein lipase (LPL) via evaginations through the basement membrane and can express costimulatory molecules under inflammatory conditions [31]. The primed CD4+ T-cells would then recirculate to the lamina propria [32, 33], and subsequent contact with gluten would induce their activation and proliferation, with production of proinflammatory cytokines, such as TNF-*α*, IL-1*β*, IL-6, IL-15, IL-17A, IL-17F, IL-21, IL-22, IL-23, and IL-26 (Figure 1) [34, 35]. This would result in synthesis and release of metalloproteases MMP-1, MMP-3 [36], and Keratinocyte Growth Factor (KGF) by stromal cells [37], which would induce cryptal hyperplasia [38]. The next stage, villous atrophy, would be due to enterocyte death induced by IELs [39, 40]. In fact CD8+ IELs from CD patients have been shown to respond to gluten peptides presented by HLA-A2 [41]. Additionally, there is an overexpression of membrane bound IL-15 on enterocytes in active CD which induces the expression of the NK receptors CD94 [13] and NKG2D by CD3+ IELs. The ligand for NKG2D and MIC-A is overexpressed on enterocytes in active CD, and this supports the involvement of the MIC-A/NKG2D pathway in the epithelial atrophy of CD [12].

## 4. Association between Microbiota and Dietary Factors in Development of Innate and Adaptive Immunity

Gut microbiota is the collection of microbial populations that reside in the gastrointestinal tract. It is characterized by an interplay between different cells and their defense systems, food particles, molecules derived from digestion, and the vast array of residing microbial species with their secretory products. These microorganisms present in the gut lumen form the microbiota that performs several physiological functions, that is, the absorption and digestion processes, tolerance to non-self-food antigens, and defense from pathogens.

The composition of main bacterial populations does not stabilize until after the first few years of life. In this period, the microbiota gradually colonizes the mucosal and skin surfaces of the neonate and exerts the greatest effect on the development of the immune system [42]. Components of the intestinal microbiota play a crucial role in the postnatal development of the immune system. During the early postnatal period, the intestinal microbiota stimulates the development of both local and systemic immunity, while later on these components evoke inhibitory regulatory mechanisms intended to keep both mucosal and systemic immunity in check [43]. In this postnatal period, components of the normal microbiota induce a transient physiological inflammatory response in the gut associated with enlargement of the mucosal-associated lymphatic tissue and increases in its cellularity [43, 44]. Many studies have shown that microbial colonization of animals living in germ-free conditions results in an increase in immunoglobulin levels, the production of specific antibodies, and substantial changes in mucosal-associated lymphocyte tissues and cell populations [45, 46]. Interestingly, the microbial colonization of germ-free mice also speeds up the biochemical maturation of enterocytes, resulting in a shift in the specific activities of brush-border enzymes nearly to the extent found in conventional mice. Moreover, a similar introduction of microorganisms alters the synthesis of sugar chains in membrane-associated glycoproteins, which could influence the gut barrier function [47–49]. In some cases, impaired function of the intestinal barrier leads to an increase in antibodies directed against antigens present in the intestinal lumen. It was recently shown that the appearance of these antibodies or/and autoantibodies in individuals lacking clinical symptoms may have important predictive value for the development of inflammatory and autoimmune diseases [50, 51]. In the case of autoimmune diseases, considerable effort has been made to understand mechanisms leading to the loss of self-tolerance.

A principal function of the microbiota is to protect the intestine against colonization by exogenous pathogens and potentially harmful indigenous microorganisms. However, in certain conditions, some species of bacteria are thought to be capable of causing disease by producing inflammation, infection, or increasing cancer risk for the host [52]. Infectious agents are considered possible environmental factors triggering autoimmune diseases. Particular bacterial populations that are typically found in very low abundance can acquire pathogenic properties. These conditions include inherent immune defects as well as changes in diet and/or acute inflammation and can result in the disruption of the normal balanced state of the gut microbiota, which is referred to as dysbiosis [53]. Dysbiosis involves the abnormal accumulation or increased virulence of certain commensal populations of bacteria, thereby transforming former symbionts into “pathobionts.” Pathobionts are typically colitogenic and can trigger intestinal inflammation [52]. The breakdown of the normal microbial community contributes to increase in the risk of pathogen infection and the overgrowth of harmful pathobionts and inflammatory disease.

The intestinal dysbiosis of CD patients is characterized by increases in numbers or proportions of* Bacteroides* spp. and reductions in those of* Bifidobacterium* spp. and* B. longum*, which were not completely normalized after patient adherence to a gluten-free diet (GFD) [54–56].* E. coli* and* Staphylococcus* numbers were also higher in feces and biopsies of untreated CD patients than in those of controls, but the differences were normalized after gluten withdrawal [55]. The analyses of the prevalence of bacterial species associated with duodenal biopsies also revealed a reduction in* Bacteroides* species diversity in patients, untreated and treated with the GFD, in comparison with controls [57].

In addition to dysbiosis, infections may also disrupt the intestinal homeostasis leading to chronic inflammation and tissue damage, which could eventually contribute to reduced gluten tolerance [58]. In fact, infections have often been considered to initiate the process in genetically predisposed individuals. Infections can influence the host's immune tolerance by different mechanisms. These include polyclonal lymphocyte activation, increased immunogenicity of organ autoantigens secondary to infection-mediated inflammation, or antigen mimicry molecular mechanisms [59]. One major hypothesis explaining how infectious components can cause autoimmune reactions is based on the concept of cross-reactivity, also known as “molecular mimicry,” that is the similarity between the epitopes of autoantigens and epitopes of harmless environmental antigens [60]. The adjuvant activity of microbial components may participate in the stimulation of APCs, such as dendritic cells, that leads to the abnormal processing and presentation of self-antigens. Homeostasis of the intestinal mucosa may be disturbed by pathogenic microorganisms and toxins attacking the intestine or by inadequately functioning components of the immune system, as observed in immunodeficiency or in cases of dysregulated mechanisms of the mucosal immune system. The intestinal mucosa can be affected as a consequence of either insufficient activity or exaggerated activation of the immune system [61]. Various complex diseases may occur as a consequence of disturbances of mucosal barrier function or of changes in mechanisms regulating mucosal immunity to food or component of the microbiota [62, 63]. The complexity and interindividual variation of the gut microbiota composition in humans represent a confounding factor in the efforts to determine the possible significance of individual commensal microbial organisms in disease pathogenesis.

Recent data have described a possible link between CD onset in susceptible patients and diverse infectious agents, which may have occurred as early as during the perinatal period. Plot et al. [64] demonstrated a possible protective role that infections with EBV, CMV, and Rubella may have on susceptible individuals; it seems that encountering certain infections may establish a particular immunological background that disfavours the evolution of autoimmune conditions. Moreover, several intestinal viral triggers including adenovirus, hepatitis C virus (HCV), and rotavirus and bacterial infections capable of initiating or augmenting gut mucosal responses to gluten were suggested to play a role in the pathogenic mechanism of CD [65]. An environmental factor, such as an infectious agent, is thought to precipitate the disease, via various pathogenic mechanisms, such as molecular mimicry, resulting in modulation of the host's immune tolerance. Several other gastrointestinal pathogens have been associated with the development of CD, with varying outcomes; most are isolated case reports. Other pathogens such as* Campylobacter jejuni*,* Giardia lamblia*,* Rotavirus* infection, and* Enterovirus* infection have also been associated with development of CD [65].

Abnormal components found among the microbial inhabitants adhering to the diseased jejunal mucosa have been described and recently analyzed using new microbiological methods [66]. Bacteria were identified by 16S rDNA sequencing in DNA extracted from biopsies. Profound changes in the fecal and duodenal microbiota composition of patients with active disease who are on a gluten-free diet have also been demonstrated [56].* Bacteroides* and* Clostridium leptum* groups were more abundant in faeces and biopsies of CD patients than in controls regardless of the stage of the disease.* Escherichia coli* and* Staphylococcus* counts were also higher in faces and biopsies of nontreated CD patients than in those of controls. Interestingly, some commensal bacteria, such as* Escherichia coli*, promoted the activation of innate immune cells by gliadin, whereas others such as* Bifidobacteria* exerted inhibitory effects [67]. Dietary factors seem particularly relevant at early stages when the immature neonate's gut is acquiring and shaping its own microbiota and undergoing major physiological and immunological developments up to the point when the immune system acquires full competence and tolerance to nonharmful antigens [68]. The period in which the human host is most acutely influenced by the microbiota is the postnatal period, during which the germ-free neonate moves from the sterile environment of its mother's uterus into a world full of microorganisms and during which the neonate's mucosal and skin surfaces become gradually colonized. Particularly relevant seems to be the era in which we introduce the gluten in the diet. As early as the seventies, it had become clear that the introduction of gluten in the diet after the fourth month of life would reduce the incidence of CD onset [69]. Infants who carry either the HLA-DR3 or DR4 alleles or who have a first-degree relative affected by CD [70] have a fivefold increased risk of developing CD autoimmunity with the presentation of positive tissue transglutaminase (tTG) autoantibody if they are exposed to gluten in the first three months of life. Infants introduced to gluten at 7 months or later also had an increased risk of CD compared with those exposed between 4 and 6 months.

Some explanations have been reported by Norris et al. [70] for the increased risk of CD when first gluten exposure occurs in younger and older children instead of at the age of 4–6 months. On the one hand, in younger children, early introduction of solid foods (i.e., before the intestinal immune system reaches a certain level of maturation) may lead to intolerance [71]. The increased risk of CD in children introduced to gluten at 7 months or older might be due to the larger amounts of gluten intake at the first exposure [70]. The ESPGHAN Committee on nutrition has outlined possible practical suggestions on the introduction of complementary feeding to avoid both early (<4 months) and late (≥7 months) introduction of gluten and to gradually introduce small amounts of gluten whilst the infant is still breast-fed [72] in order to reduce the predisposition to CD later in life [73]. However, the time of first exposure to potentially allergenic foods in infants differs significantly between countries and occurs much earlier than recommended in some countries, as reported in [74]. A recent meta-analysis of observational retrospective studies was used to analyze the protective role of breast-feeding against CD onset and concluded that increased duration of breastfeeding is associated with a reduced risk of CD [75]. A study of 627 cases with confirmed CD revealed that the risk of CD was reduced in a group of children aged <2 years if they were still being breast-fed when dietary gluten was introduced and the risk increased when the gluten was introduced in the diet in large amounts [76].

It is also important to examine further whether favorable infant dietary patterns postpone CD onset or in fact reduce the overall lifetime risk of the disease [76]. Other recent studies have pointed to the role of breast-feeding in delaying CD in infancy. D'Amico et al. [77] showed that children with CD who had been exclusively breast-fed had a delayed onset and less severe disease symptoms than those who had not been exclusively breast-fed [77]. In spite of all the evidence reported, it remains unclear whether those children breast-fed during the introduction of gluten are more likely to develop an extraintestinal (atypical) CD [78]. A series of 162 celiac children registered by the University of Chicago revealed that children breast-fed at the time of gluten introduction were just as likely to develop both intestinal and extraintestinal symptoms, whereas children who were not breast-fed when weaned with gluten had a much higher chance of developing intestinal symptoms [79]. Human milk provides many bioactive factors, including antimicrobial and anti-inflammatory agents, enzymes, hormones, and growth factors, many of which are involved in gut maturation and development of the infant's innate and acquired immunity [80]. Breast-feeding might affect tolerance induction in infants because of the possible transfer of small amounts of gluten and gluten-specific IgA antibodies through breast milk and the presence of factors in breast milk that affect immune system maturation and responses [81]. Also, it seems that the lymphocyte subset profile of breast-fed infants is associated with a better response to gliadin after gluten introduction. Other reasons that could explain a protective effect of breast-feeding against CD development could be related to the role human milk plays in defining microbiota composition [82] and in the incidence of infections.

There is evidence that microbiota may enhance innate immunity to pathogens. The interaction between microbiota and innate immunity develops in the gut mucosa. In particular, innate immunity cells, located in the lamina propria, promote immunological unresponsiveness to commensal bacteria, which is important for maintaining gut homeostasis. Specifically, gut-resident phagocytes are hyporesponsive to microbial ligands and commensal bacteria, and they do not produce biologically significant levels of proinflammatory molecules upon stimulation. However, the microbiota is essential for upregulating the production of pro-IL-1*β*, the precursor to IL-1*β*, in resident innate immunity cells. It has been shown that IL-1*β* may have a protective role in intestinal immunity. This role is, at least partly, mediated by its ability to induce the expression of endothelial adhesion molecules that contribute to neutrophil recruitment and pathogen clearance in the intestine [52].

Initiation of the innate immune response in the intestine is triggered by pathogen-recognition receptors (PRRs). These PRRs serve as sensors of pathogen-associated molecular patterns (PAMPs) from the intestinal lumen. The most studied PRRs are the Toll-like receptors (TLRs). TLRs are transmembrane proteins that are typically expressed by intestinal epithelial cells either on the cell surface or in endosomes. TLR signaling in the intestine is involved in epithelial cell proliferation, immunoglobulin A (IgA) production [83], and antimicrobial peptide expression, functions that are crucial for maintaining a healthy epithelial barrier [84]. PRRs are also expressed by other immune cells in the lamina propria and they can activate an inflammatory response involving both innate and adaptive immune system [85]. The intestinal barrier regulates intestinal homeostasis throughout innate and adaptive immune responses. Unfortunately, in some cases the innate immune system's attempts to protect the host fail and chronic inflammation and intestinal autoimmunity occur, such as in the case of celiac disease and IBD [86].

Recent experimental data demonstrated the existence of a strong and direct interaction between TLRs and intestinal microbiota. For example, Cheng et al. demonstrated that pediatric CD patients have lower duodenal expression of TLR2 and higher expression of TLR9 as compared to healthy controls confirming that microbiota may have a role in CD [87].

Higher TLR9 does not directly correlate to microbiota. In fact, Cheng and colleagues concluded that the overall composition, diversity, and the estimated microbe associated molecular pattern (MAMP) content of microbiota were comparable between CD and healthy subjects, but a subpopulation profile comprising eight genus-like bacterial groups was found to differ significantly between healthy subjects and CD. In healthy subjects, increased TLR2 expression was positively correlated with the expression of tight junction protein ZO-1. In CD, the expression of IL-10, IFN-gamma, and CXCR6 was higher as compared to healthy subjects.

Other recent data [87] comparing the composition of microbiota between CD patients and healthy controls demonstrated that TLRs activation by intestinal microbiota may determine different effects on the human gut of CD patients and healthy controls. These data suggest that microbiota and altered expression of mucosal receptors have a role in CD. In CD subjects, the increased expression of IL-10 and IFN-gamma may have partly resulted from the increased TLR9 expression and signaling [88].

Furthermore, recently Eiro et al. [89] studied the expression of TLRs and cytokines and revealed that they occur in duodenal mucosa and are elevated in both children and adult celiac patients. In particular, TLR4 expression was increased twofold in CD patients compared to controls. CD patients with high levels of TLR4 also showed high levels of proinflammatory cytokines (IL-1, IL-6, IL-8, and IL-17) as well as transcription factors (IRAK4, MyD88, and NF-*κ*B) [89]. These data support the hypothesis that a unique pattern of TLR expression is associated with CD independently of age at diagnosis. The evidence that pediatric and adult patients have a similar inflammatory profile will encourage treatment of both with the same immunological therapy in the future.

## 5. Dietary and Microbiota Changes and Immunomodulatory Approaches in Celiac Disease: Translational Applications

The only approved treatment for CD is the lifelong complete exclusion of the gluten from the diet. But, dietary compliance is difficult to achieve in several patients for several reasons. Indeed, we need novel strategies, targeting various factors at different levels in CD pathogenesis.

For example, the reduction of gluten antigenicity and/or elimination of toxic peptides from gluten could be walkable; in this manner, the food gluten would not have the negative effect on activation of host immune system and the consequential gut inflammation. These modifications of gluten antigenicity may be achieved in several manners, from the selection of natural cereal cultivars to the genetic modification of gluten peptide sequences or more likely producing genetically modified organisms, in which toxic sequences are deleted or silenced [90].

The other translational strategy of CD therapy derives from the knowledge of immunomodulatory mechanisms of disease. In fact, in CD, food gluten peptides activate lamina propria T-cells to produce inflammatory cytokines leading to tissue destruction. Thus, a way to block this pathogenetic event in CD could be a specific modulation of gluten reactive pathogenetic target T-cells. This modulation in gluten related to T-cell reactivity may be borrowed from the actual evidences on other immune hypersensitivity related diseases, such as allergy and autoimmune diseases. In fact, in these diseases, such as in CD, there is a pivotal role of CD4+ T-helper cells in driving and orchestrating gut inflammation and tissue destruction. The goal of pathogenetic translational therapy may be to develop vaccines consisting of synthetic peptides representing T-cells epitopes that may have the function to restore the balance between inflammatory and regulatory responses to the causative antigens [91, 92]. In CD, this could be achieved using a therapeutic vaccination constructed with the panel of most immunodominant gluten epitopes. The efficacy of vaccine therapy has been just demonstrated in allergy in which it is able to reduce allergen sensitivity and improve regulatory T-cell function.

Furthermore, there are recent findings in the alterations of the microbiota in various medical conditions, such as CD, inflammatory bowel disease, allergy, and colorectal cancer. Even though changes of the microbiota could be linked to the etiopathogenesis of these diseases, further studies are needed to understand how to modulate microbiota to ameliorate the morbidity of CD.

## 6. Conclusion

Chronic inflammatory diseases have multifactorial etiologies that involve environmental components and many immune and genetic factors. CD represents a particularly informative model for chronic inflammatory diseases. An environmental factor that precipitates disease is known (gluten); the HLA molecules that confer predisposition to the disease have been identified (HLA-DQ2/HLA-DQ8); and access to the small intestine is simple. Thus, understanding the interaction of the microbiota with pathogens and host might provide new insights into the pathogenesis of disease, as well as novel avenues for preventing and treating intestinal and systemic disorders [52].

The high increase in incidence of autoimmune disorders cannot be explained only by genetic drift and is thought to be the result of changes in the environmental factors and in their complex interaction with innate and adaptive immunity. Although there is strong support for the role of microorganisms as triggers of innate immune activation, there is no evidence of a role for molecular mimicry in CD. In other words, we do not have any evidence so far that CD pathogenesis is the result of a T-cell response against a microbial peptide cross-reacting with gluten [93].

The gut microbiota has been studied for more than a century; however, we have only recently begun to understand the ever-expanding roles for these microscopic organisms in health and disease. Despite the complexity of the gut microbiota, there is a delicate balance in bacterial populations such that any disruption in this balance leads to dysbiosis and, consequently, to decreased resistance to pathogen colonization, to the favoured growth of pathobionts, and to pathological both innate and adaptive immune responses [52]. So, in genetically predisposed individuals, gluten in association with microbial antigens can stimulate and modulate innate and adaptive immune response, sustaining a chronic mucosal inflammation, underlining this chronic disease.

In summary, CD may be considered as a model to explain the pathogenesis of several other autoimmune diseases. In particular, how in the steady state condition homeostasis is maintained due to the complete balance between pro- and anti-inflammatory factors whereas, during disease, this balance is altered. The breakdown of the interaction among microbiota, innate immunity, and genetic and dietary factors leads to the disruption of homeostasis leading to inflammation and tissue damage. Thus, focusing the attention on this interaction and its breakdown may allow a better understanding of the CD pathogenesis and finally get novel translational avenues for preventing and treating this widespread disease.
